# XDR-TB in South Africa: Theory and Practice

**DOI:** 10.1371/journal.pmed.0040163

**Published:** 2007-04-24

**Authors:** Jason Andrews, Sanjay Basu, David Scales, Duncan Smith-Rohrberg Maru, Ramnath Subbaraman

Singh and colleagues [1] highlight safeguards against the spread of XDR-TB and suggest “involuntary detention” as a key infection control measure. Yet several important elements of the current response to XDR-TB may make the application of enforced confinement ineffective and inappropriate as part of the initial response to this problem.

One irony of this discussion is that patients diagnosed with drug-resistant TB in KwaZulu-Natal are being turned away from the referral hospitals where second-line therapy takes place. There is a waiting list of more than 70 patients for admission to King George V Hospital, where the majority of MDR-TB therapy is provided. Rather than keeping patients “in”—the debate posed in this article—the reality is that health services are unable to accommodate the burden of MDR-TB patients seeking care.

The authors cite United States policies during MDR-TB outbreaks as evidence of the success of detention but fail to note that US confinement measures were rarely invoked. The New York City Tuberculosis Working Group concluded: “It is unethical, illegal, and bad public health policy to detain ‘noncompliant’ persons before making concerted efforts to address the numerous systemic deficiencies that make adherence to treatment virtually impossible” [2]. Thus, patients were first offered directly observed therapy as outpatients. Among the few patients cited as non-adherent, less than half were detained. Monetary incentives and transportation vouchers were provided for outpatients, as well as housing to the homeless [3]. In contrast, many MDR-TB patients in KwaZulu-Natal must travel several hours monthly to receive treatment.

It is estimated that the South African government will spend 15 billion rand (~US$1.9 billion) for the upcoming World Cup, much of it for building stadiums [4]. Yet, while the largest outbreak of XDR-TB ever recorded is unfolding, little appropriate investment has been made. Purchasing trailer homes as isolation facilities, providing particulate respirator masks in all hospitals, and instituting other basic infection control procedures is immediately necessary. Framing the debate about forced confinement in terms of individual liberty versus threat to society neglects the true injustice taking place.

While Singh and colleagues discuss the importance of “reciprocity,” they fail to mention the most important reciprocity obligation of those instituting confinement: providing the proper standard of medical care to detained patients. At present, many XDR-TB patients are provided therapy that includes only two active agents—a recipe for amplification of resistance. While XDR-TB patients elsewhere have been successfully treated with other regimens [5], the majority of South African patients have yet to access many second-line drugs, including capreomycin, moxifloxacin, para-aminosalicylic acid, or adjunctive thoracic surgery. Without these, they are left to die without a fighting chance, two years after this outbreak was first reported. The intent of detention in the US was to provide short inpatient stays and curative therapy. The median period of detention was three weeks, and only 2% of patients died from tuberculosis [3]. In South Africa, XDR-TB is nearly universally fatal under current treatments, and detention would presumably be sustained until death. Our willingness to respond to the realities of patient needs, rather than to abstract theories, will determine the success of the response to XDR-TB.
